# Complete Genome Sequence of *Clavibacter michiganensis* subsp. *insidiosus* R1-1 Using PacBio Single-Molecule Real-Time Technology

**DOI:** 10.1128/genomeA.00396-15

**Published:** 2015-05-07

**Authors:** You Lu, Deborah A. Samac, Jane Glazebrook, Carol A. Ishimaru

**Affiliations:** a Department of Plant Biology, University of Minnesota, St. Paul, Minnesota, USA; b Microbial and Plant Genomics Institute, University of Minnesota, St. Paul, Minnesota, USA; c USDA-ARS Plant Science Research Unit, St. Paul, Minnesota, USA; d Department of Plant Pathology, University of Minnesota, St. Paul, Minnesota, USA

## Abstract

We report here the complete genome sequence of *Clavibacter michiganensis* subsp. *insidiosus* R1-1, isolated in Minnesota, USA. The R1-1 genome, generated by a *de novo* assembly of PacBio sequencing data, is the first complete genome sequence available for this subspecies.

## GENOME ANNOUNCEMENT

*Clavibacter michiganensis* subsp. *insidiosus* is a Gram-positive coryneform bacterium which causes bacterial wilt of alfalfa (*Medicago sativa* L.), the most widely cultivated forage legume (1). Bacterial wilt of alfalfa occurs throughout most alfalfa-growing regions worldwide. For this reason, the pathogen is considered a phytosanitary risk for the international seed movement. *C. michiganensis* subsp. *insidiosus* R1-1 was isolated from stem tissue of a *Medicago truncatula* plant inoculated with ground roots of symptomatic alfalfa plants from disease nurseries at the University of Minnesota Rosemount Research and Outreach Center in Rosemount, MN.

There are six subspecies of *C. michiganensis* identified as plant pathogens (2, 3). Complete genome sequences are currently available for a single strain of three subspecies: the tomato pathogen, *C. michiganensis* subsp. *michiganensis* (4), the potato pathogen, *C. michiganensis* subsp. *sepedonicus* (5), and the corn pathogen, *C. michiganensis* subsp. *nebraskensis* (2). Comparative genome analyses revealed extensive rearrangement between the genomes and the presence or absence of genes encoding known virulence factors (2, 5). Previous research on *C. michiganensis* subsp. *insidiosus* has focused primarily on plant resistance and diagnostic methods (6, 7). The lack of a complete genome sequence has hampered progress in understanding the molecular mechanisms of host-pathogen interactions.

An earlier *de novo* assembly of the *C. michiganensis* subsp. *insidiosus* R1-1 genome from paired-ended reads from Illumina GAIIx system sequencing resulted in 103 contigs containing ambiguous bases due to the presence of repetitive elements and a high G+C genome content. Here, we utilized PacBio single-molecule real-time (SMRT) sequencing technology (8) to generate a *de novo* assembly of the complete genome sequence of *C. michiganensis* subsp. *insidiosus* R1-1. Genomic DNA extracted from *C. michiganensis* subsp. *insidiosus* R1-1 was prepared as a 20-kb library for P6-C4 chemistry, followed by BluePippin size selection at 15 kb. The PacBio RSII sequencing system generated 64,530 reads, with a mean read length of 10,432 bp from one SMRT cell. The initial assembly was conducted using Hierarchical Genome Assembly Process 3 (HGAP3) (9) in PacBio SMRT portal version 2.2.0, and four contigs were assembled, with a mean coverage of 163.44×. This assembly was corrected with the Quiver consensus algorithm to obtain a high-accuracy genome assembly (9). Further improvement of the quality of the genome sequence was performed with Pilon (10) using data generated by Illumina GAIIx platform sequencing. This process corrected 43 indel errors in the PacBio assembly. The resulting genome assembly contains four circular contigs, corresponding to one chromosome (3,207,520 bp; 72.96% G+C content) and three circular plasmids, pCI1 (47,690 bp; 67.78% G+C content), pCI2 (49,401 bp; 67.58% G+C content), and pCI3 (103,451 bp; 66.16% G+C content).

Annotation was performed with the NCBI Prokaryotic Genome Annotation Pipeline (http://www.ncbi.nlm.nih.gov/genome/annotation_prok/). In total, 3,012 protein-coding genes, two rRNA operons (16S, 23S, 5S), 46 tRNA genes, and 27 copies of the insertion element IS*1122* were identified in the R1-1 genome.

The availability of the complete genome sequence of *C. michiganensis* subsp. *insidiosus* R1-1 will be valuable for further comparative genomic analyses and the development of molecular diagnostic tools.

### Nucleotide sequence accession numbers.

The *C. michiganensis* subsp. *insidiosus* R1-1 genome sequence was deposited in GenBank under the accession numbers CP011043 to CP011046. The versions described here are the first versions.
